# SARS-CoV-2 and influenza coinfection throughout the COVID-19 pandemic: an assessment of coinfection rates, cohort characteristics, and clinical outcomes

**DOI:** 10.1093/pnasnexus/pgac071

**Published:** 2022-07-04

**Authors:** Colin Pawlowski, Eli Silvert, John C O'Horo, Patrick J Lenehan, Doug Challener, Esteban Gnass, Karthik Murugadoss, Jason Ross, Leigh Speicher, Holly Geyer, A J Venkatakrishnan, Andrew D Badley, Venky Soundararajan

**Affiliations:** nference, Cambridge, MA 02139, USA; nference, Cambridge, MA 02139, USA; Mayo Clinic, Rochester, MN 55902, USA; nference, Cambridge, MA 02139, USA; Mayo Clinic, Rochester, MN 55902, USA; nference, Cambridge, MA 02139, USA; nference, Cambridge, MA 02139, USA; nference, Rochester, MN 55902, USA; Mayo Clinic, Jacksonville, FL 32224, USA; Mayo Clinic, Scottsdale, AZ 85259, USA; nference, Cambridge, MA 02139, USA; Mayo Clinic, Rochester, MN 55902, USA; nference, Cambridge, MA 02139, USA; nference, Rochester, MN 55902, USA

**Keywords:** Flurona, COVID-19, SARS-CoV-2, Influenza, Flu, Co-infection

## Abstract

Case reports of patients infected with COVID-19 and influenza virus (“flurona”) have raised questions around the prevalence and severity of coinfection. Using data from *HHS Protect Public Data Hub, NCBI Virus*, and *CDC FluView*, we analyzed trends in SARS-CoV-2 and influenza hospitalized coinfection cases and strain prevalences. We also characterized coinfection cases across the *Mayo Clinic Enterp*rise from January 2020 to April 2022. We compared expected and observed coinfection case counts across different waves of the pandemic and assessed symptoms and outcomes of coinfection and COVID-19 monoinfection cases after propensity score matching on clinically relevant baseline characteristics. From both the Mayo Clinic and nationwide datasets, the observed coinfection rate for SARS-CoV-2 and influenza has been higher during the Omicron era (2021 December 14 to 2022 April 2) compared to previous waves, but no higher than expected assuming infection rates are independent. At the Mayo Clinic, only 120 coinfection cases were observed among 197,364 SARS-CoV-2 cases. Coinfected patients were relatively young (mean age: 26.7 years) and had fewer serious comorbidities compared to monoinfected patients. While there were no significant differences in 30-day hospitalization, ICU admission, or mortality rates between coinfected and matched COVID-19 monoinfection cases, coinfection cases reported higher rates of symptoms including congestion, cough, fever/chills, headache, myalgia/arthralgia, pharyngitis, and rhinitis. While most coinfection cases observed at the Mayo Clinic occurred among relatively healthy individuals, further observation is needed to assess outcomes among subpopulations with risk factors for severe COVID-19 such as older age, obesity, and immunocompromised status.

Significance Statement Reports of COVID-19 and influenza coinfections (“flurona”) have raised concern in recent months as both COVID-19 and influenza cases have increased to significant levels in the United States. Here, we analyze trends in coinfection cases over the course of the pandemic to show that these coinfection cases are expected given the background prevalences of COVID-19 and influenza independently. In addition, from an initial analysis of these coinfection cases, which have been observed at the Mayo Clinic, we find that these coinfection cases are extremely rare, have mostly been observed in relatively young, healthy patients, and do not have an increased risk of hospitalization, ICU admission, or death while they do have more emblematic viral symptoms.

## Introduction

Since the beginning of the COVID-19 pandemic there have been lingering concerns around the possibility of a “twindemic” with influenza (1), particularly as the COVID-19 pandemic extends through influenza seasons. Amidst the ongoing surge of Omicron-associated COVID-19 cases, recent reports of patients testing positive for both COVID-19 and influenza, dubbed as “flurona” patients (2), have raised an alarm. Coinfection with SARS-CoV-2 and influenza has been reported since early in the pandemic. A meta-analysis of coinfection prevalence studies from December 2019 to September 2020 found 79 individuals with concurrent COVID-19 and influenza infection among a total of 3,070 COVID-19 cases (3). Some studies have reported that up to 20% of COVID-19 cases can demonstrate coinfection with other respiratory viruses (4). However, there is a lack of understanding on whether a monoinfection of SARS-CoV-2 or influenza makes one more susceptible to secondary viral infection and whether the disease severity of two concurrent infections is greater than a single infection.

Epidemiological data on SARS-CoV-2, influenza, and coinfection-related hospitalizations in the United States is available from the Health and Human Services (HHS) Protect Public Data Hub (5). The HHS Protect Public Data Hub is a central COVID-19 data repository, which aggregates the United States healthcare data from various HHS operating divisions including: Centers for Disease Control and Prevention (CDC), Centers for Medicare and Medicaid Services (CMS), Health Resources and Services Division (HRSD), and others. Availability of this national-level data allows us to investigate the temporal trends of COVID-19 and influenza coinfections. Other data repositories such as NCBI Virus (6) and FluView (7) provide valuable data on SARS-CoV-2 and influenza strain prevalence in the United States. In addition, understanding the clinical characteristics and outcomes associated with COVID-19 and influenza coinfections requires a longitudinal analysis of data from sources such as clinical trials or electronic health records (EHRs), which have patient-level information. Previously, analyses of EHR data from the Mayo Clinic Enterprise have been used to assess various characteristics of COVID-19 symptomology (8), duration of infection (9), and COVID-19-associated complications (10, 11).

In this study, we analyze epidemiological data from HHS Protect Public Data Hub in order to evaluate trends in COVID-19, influenza, and coinfection cases across the entire United States in relation to trends in SARS-CoV-2 and influenza strain prevalences. In addition, we apply machine-augmented curation models (neural networks) and other techniques to conduct an observational study over the Mayo Clinic Enterprise EHR database to assess the prevalence and clinical characteristics of coinfections with SARS-CoV-2 and influenza viruses in this multistate health system.

## Methods

### Tracking temporal and geographic trends in SARS-CoV-2 and influenza coinfection-related hospitalizations in the United States

In order to track temporal trends in SARS-CoV-2, influenza, and coinfection-related hospitalizations in the United States over the course of the pandemic, we used the *“COVID-19 Reported Patient Impact and Hospital Capacity by Facility*” dataset (12). The hospital population includes all hospitals registered with CMS as of 2020 June 1. For each reporting hospital, this dataset includes 7-day average case counts of hospitalized patients with PCR-confirmed COVID-19, PCR-confirmed influenza, and coinfections. We considered a subset of these hospitals with data available for these variables each week from 2020 October 30 to 2022 April 2. In addition, we filtered out hospitals with inconsistent data (e.g. more coinfection hospitalized cases than total influenza hospitalized cases for a single week) to obtain a final set of 1,377 hospitals for analysis. Since case counts less than four individuals are censored in this dataset, we imputed these values to be 1 for the computation of averages. For each week, we summed the average case counts across all 1,377 hospitals to obtain estimates for the average number of hospitalized patients with SARS-CoV-2, influenza, and coinfections at these facilities during that week.

### Mayo Clinic Institutional Review Board

We performed a retrospective analysis on EHR data from the Mayo Clinic under the IRB #20–003278 *“Study of COVID-19 patient characteristics with augmented curation of Electronic Health Records (EHR) to inform strategic and operational decisions.”* This includes all individuals with research authorization on file who received a PCR test for SARS-CoV-2 at the Mayo Clinic sites since 2020 January 1. The Mayo Clinic Enterprise is a multistate academic medical center with major campuses in Rochester, MN, Jacksonville, FL, and Scottsdale, AZ, along with additional satellite sites in other states including Iowa and Wisconsin.

### Cohort definitions

The study population included all individuals who have received a positive PCR test for SARS-CoV-2 at the Mayo Clinic between 2020 January 1 and 2022 April 2. From this study population, we constructed the following cohorts: (1) “Overall COVID-19”: all individuals with a positive PCR test for SARS-CoV-2 and (2) “COVID-19 + Flu”: all individuals with a positive PCR test for SARS-CoV-2 along with a diagnosis of influenza within 14 days.

By definition, all individuals in the study population were included in the “Overall COVID-19” cohort. In addition, some individuals in the study population were included multiple times in this cohort if they had multiple positive PCR tests for SARS-CoV-2 during the study period spaced at least 90 days apart. For each individual, the date of the first positive SARS-CoV-2 PCR test was considered to be the primary infection, and the date of each subsequent positive SARS-CoV-2 PCR test spaced at least 90 days apart was considered to be a reinfection.

To construct the “COVID-19 + Flu” cohort, we used a combination of data sources from within the Mayo Clinic Enterprise to determine influenza diagnosis: laboratory tests (Table S1, Supplementary Material), diagnostic billing codes (Table S2, Supplementary Material), and unstructured clinical notes. Cases without a laboratory correlate were manually reviewed to confirm influenza diagnosis. If any of these data sources indicated that an individual in the “Overall COVID-19” cohort had an influenza diagnosis within +/−14 days of their positive SARS-CoV-2 PCR test date, then this individual was included in the “COVID-19 + Flu” cohort. If there was conflicting information across the data types, i.e. there was a negative influenza lab test result within +/−7 days of a diagnostic billing code or diagnosis based on the notes, this was not considered to be a case of coinfection. Similar to the “Overall COVID-19” cohort, individuals may be included multiple times in the “COVID-19 + Flu” cohort if they had multiple co-occurrences of SARS-CoV-2 and influenza infection. The methodology to determine influenza diagnoses from the unstructured clinical notes is described below.

### Augmented curation to determine influenza diagnosis from clinical notes

To identify influenza diagnoses in the clinical notes, we used a neural network-based natural language processing (“augmented curation”) algorithm that classifies the sentiment of mentions of influenza in the clinical notes of the overall COVID-19 cohort. In particular, we applied a BERT-based phenotype sentiment model, which has previously been used to determine signs and symptoms of COVID-19 (8). For each occurrence of a phenotype in a clinical note, this model outputs one of the following labels: “Yes”: confirmed diagnosis of the phenotype, “Maybe”: uncertain/differential diagnosis of the phenotype, “No”: ruled-out diagnosis of the phenotype, or “Other”: all other mentions of the phenotype (e.g. family history). This model was developed by fine-tuning a SciBERT model on a set of 18,490 manually annotated sentences from clinical notes in the Mayo Clinic EHR including almost 250 different cardiovascular, pulmonary, and metabolic diseases and phenotypes. For positive and negative sentiment classification tasks, this model achieves an out-of-sample accuracy of 93.6% with recall and precision values above 95%.

We ran this phenotype sentiment model on all sentences that contained a mention of any of the following synonyms of influenza: “influenza,” “influenza A,” “influenza B,” “influenza A/B,” “influenza virus,” “flu,” “avian flu,” “H1N1,” “H5N1,” “H3N2,” and “H7N9.” Since each of these phenotypes were masked prior to sentence tokenization for the classification task, we did not augment the tokenizer for this use case. Individuals with at least one clinical note labeled “Yes” by the model with ≥ 90% Confidence Interval (CI) within +/−14 days of their positive SARS-CoV-2 PCR test were flagged as positive for influenza. Cases without laboratory correlates were manually reviewed and confirmed for inclusion in the “COVID-19 + Flu” cohort.

### Curation of clinical covariates from structured EHR data

For each cohort, we curated clinical covariates from the structured EHR data with features including: demographics (age, sex, race, and ethnicity), geographic location and time of COVID-19 PCR test, COVID-19 vaccination status, influenza vaccination status, and comorbidities, and 30-day clinical outcomes including: hospitalization, ICU admission, and mortality. For each individual case in each cohort, we considered the date of the positive PCR test for SARS-CoV-2 to be the index date (day = 0) to define the clinical covariates. To classify the geographic location of SARS-CoV-2 PCR testing, we used the following Mayo Clinic regions: Mayo Clinic—Arizona (includes tests administered at the major campus in Scottsdale, AZ), Mayo Clinic—Florida (includes tests administered at the major campus in Jacksonville, FL), and Mayo Clinic—Midwest (includes tests administered at the major campus in Rochester, MN as well as surrounding Mayo Clinic Health Systems sites in Minnesota, Wisconsin, and Iowa).

To determine COVID-19 vaccination status, we considered the following FDA-authorized vaccines: Janssen (Ad26.COV2.S), Moderna (mRNA-1273), and Pfizer/BioNTech (BNT162b2). The categories for COVID-19 vaccination status were defined as: “Unvaccinated”: individuals who do not have an FDA-authorized COVID-19 vaccine on record, “Partial”: individuals who have received exactly one dose of either BNT162b2 or mRNA-1273, “Full”: individuals who have received exactly one dose of Ad26.COV2.S or exactly two doses of either BNT162b2 or mRNA-1273 at least 14 days prior to their index date, and “Boosted”: individuals who are fully vaccinated and have received an additional dose of either Ad26.COV2.S, BNT162b2, or mRNA-1273 at least 14 days prior to their index date. For each individual case, vaccination status is determined at the time of the index date, so it is possible that some individuals who are fully vaccinated now may be considered unvaccinated on their index dates. To determine influenza vaccination status, we checked if the individual received an influenza vaccine at least 14 days prior to their index date and within the current flu season beginning on August 1st. Since all patients in our study population had an index date but not necessarily an influenza diagnosis date, we defined influenza vaccination status relative to the index date. For example, an individual with an index date on 2021 October 15 would need to have received a flu vaccine between 2021 August 1 and 2021 October 1 in order to be considered vaccinated for the flu season. In Table S3 (Supplementary Material), we provide a comprehensive list of the influenza vaccines considered.

We considered the following time periods of SARS-CoV-2 PCR testing dates: 2020 March 12 to 2021 March 15; 2021 March 16 to 2021 June 15; 2021 June 16 to 2021 December 13; and 2021 December 14 onward. These time periods roughly correspond to the time periods when the SARS-CoV-2 strains Wuhan (original), Alpha, Delta, and Omicron were the dominant strains in the United States over the course of the study.

To determine the comorbidities for each cohort, we considered all 31 disease categories in the Elixhauser comorbidity index (13), including: congestive heart failure, cardiac arrhythmia, valvular disease, pulmonary circulation disorder, peripheral vascular disorder, hypertension (uncomplicated), hypertension (complicated), paralysis, other neurological disorder, chronic pulmonary disease, diabetes (uncomplicated), diabetes (complicated), hypothyroidism, renal failure, liver disease, peptic ulcer disease (excluding bleeding), AIDS/HIV, lymphoma, metastatic cancer, solid tumor without metastasis, rheumatoid arthritis, coagulopathy, obesity, weight loss, fluid and electrolyte disorders, blood loss anemia, deficiency anemia, alcohol abuse, drug abuse, psychoses, and depression. For each disease category, individuals with an associated ICD-10 code within the past 5 years prior to their positive SARS-CoV-2 PCR testing date were counted as positive for the phenotype. For each individual, a single Elixhauser Comorbidity Index score was calculated using the Van Walraven method (14).

### SARS-CoV-2 variant prevalence from the NCBI Virus database

We used the NCBI Virus database (6) to determine the prevalence of the different SARS-CoV-2 variants over time. This dataset allows us to find the times of dominance for each variant and evaluate the most likely SARS-CoV-2 variant contributing to coinfection cases.

From the NCBI Virus database, we retrieved 1,832,279 SARS-CoV-2 genome samples from human hosts in the United States, which were collected from 2021 January 1 to 2022 March 23 and span 912 PANGO lineages. We categorized the samples as an Alpha, Beta, Delta, Gamma, or Omicron variant using the variant classification information from the CDC to map the PANGO lineages to particular variants (15). We found the prevalence of the variants on each day in our study period by normalizing the daily variant sample count by the daily total sample count in the NCBI Virus dataset. In addition, we used this dataset to determine the dates at which a new variant became the dominant strain in the United States (i.e. the first date that Alpha was more prevalent than the ancestral lineage and the first date that Delta was more prevalent than Alpha).

### Influenza strain prevalence from the CDC FluView database

Similarly, we used the CDC FluView database (7) to determine the prevalences of the two main influenza strains (A(H1N1)pdm09 and A(H3N2)) over time. This database provides the number of influenza strain types of all those sampled from sites in the United States on a weekly basis. From this database, we retrieved 62,777 influenza samples with known subtyping from 2020 January 1 to 2022 March 23. The six strains considered were A(H3N2v), A(H1N1)pdm09, A(H3N2), B, BVIC, and BYAM. We computed the prevalence of the strains for each week in our study period as the weekly strain sample count divided by the weekly total subtype sample count.

### Estimates of COVID-19 and influenza coinfection prevalences

To estimate the prevalence of reported COVID-19 and influenza coinfections, we divided the number of reported COVID-19 and influenza cases by the total number of COVID-19 cases observed in the Mayo Clinic EHR database. In addition, to estimate the total (reported + unreported) prevalence of COVID-19 and influenza coinfections, we divided the number of observed COVID-19 and influenza coinfections (from lab tests only) by the number of COVID-19 cases with lab tests for influenza available. Finally, to estimate the prevalence of COVID-19 coinfections among all influenza cases, we divided the number of reported COVID-19 and influenza cases by the total number of influenza cases observed during the study period. For each estimate, 95% CI using Wilson's score method (16) are reported, which were computed using the “stats” package (version 4.1.2) in R.

### Estimates of the expected number of coinfection cases

For each of the study time periods, we estimated the expected number of COVID-19 and influenza coinfection cases based upon the background incidence rates of COVID-19 and influenza at the Mayo Clinic. We considered all cases at the Mayo Clinic during each study time period with PCR testing data available for both COVID-19 and influenza within +/− 14 days, including both positive and negative PCR tests. We considered the same time periods as described above: 2020 March 12 to 2021 March 15; 2021 March 16 to 2021 June 15; 2021 June 16 to 2021 December 13; and 2021 December 14 onward. For each case, the date of the SARS-CoV-2 PCR test was used to determine the time period.

For each time period, we estimated the probability of COVID-19 among the cotested population as the number of cotested cases with positive SARS-CoV-2 PCR tests divided by the total number of cotested cases. Similarly, we estimated the probability of Influenza among the cotested population as the number of cotested cases with positive PCR tests for Influenza divided by the total number of cotested cases. We compute the expected number of COVID-19 and Influenza cases for the time period as

$$\begin{equation*}
E\left[ {COVID - 19 + ,Flu + |Co - Tested} \right] =
\end{equation*}
$$

$$\begin{equation*}
{n_{co - tested}}*\Pr \left( {COVID - 19 + {\rm{ }}|{\rm{ }}Co - Tested} \right)*\Pr \left( {Flu + |Co - Tested} \right){\rm{ }}*{\rm{ }}c,
\end{equation*}
$$

where:

*n_cotested_*: total number of cotested cases during the time period,

*Pr(COVID-19+ | Co-Tested)*: probability of COVID-19 among cotested population for the time period,

*Pr(Flu+ | Co-Tested)*: probability of influenza among the cotested population for the time period, and

*c*: estimated model parameter.

In the above estimate, the main assumption is that the probabilities of testing positive for COVID-19 and influenza are independent in the general population. In addition, we assume that the estimated model parameter *c* is constant over the course of the study. For these estimates of expected coinfection cases, we compute 95% CI using the Delta method (17). In the following section, we provide a detailed description of the probability model underlying this formula along with a description of the method used to estimate the model parameter *c*.

### Probability model used to estimate the expected number of coinfection cases

In this section, we provide a description of the probability model which is used to estimate the expected number of COVID-19 and influenza coinfection cases at the Mayo Clinic. The primary assumption of this model is that the probabilities of testing positive for COVID-19 and influenza are independent in the general population of patients at the Mayo Clinic. For example, if the background prevalences of COVID-19 and influenza in the general population were 1% and 0.2% respectively, then we would expect the prevalence of coinfections to be 1% * 0.2% = 0.002%.

For each individual case, we define the index date to be the date of the SARS-CoV-2 PCR test. We define the following probability events:

*COVID-19+:* the individual received a positive PCR test for COVID-19 within +/− 14 days of the index date,

*Flu+:* the individual received a positive PCR test for influenza within +/− 14 days of the index date, and

*Co-Tested:* the individual received PCR tests for both COVID-19 and influenza within +/− 14 days of the index date.

Given these events, we define the following probabilities of COVID-19 and influenza monoinfections:

*Pr(COVID-19+)*: probability that the individual received a positive PCR test for COVID-19 within +/− 14 days of the index date (among patients with and without SARS-CoV-2 PCR testing),

*Pr(COVID-19+ | Flu+)*: probability that the individual received a positive PCR test for COVID-19 within +/− 14 days of the index date (among patients with positive PCR tests for influenza),

*Pr(COVID-19+ | Co-Tested)*: probability that the individual received a positive PCR test for COVID-19 within +/− 14 days of the index date (among patients with PCR tests available for both influenza and COVID-19),

*Pr(COVID-19+ | Flu+, Co-Tested)*: probability that the individual received a positive PCR test for COVID-19 within +/− 14 days of the index date (among patients with a positive PCR test result for influenza and a COVID-19 PCR test result available).

*Pr(Flu+)*: probability that the individual received a positive PCR test for influenza within +/− 14 days of the index date (among patients with and without PCR testing for influenza),

*Pr(Flu+ | COVID-19+)*: probability that the individual received a positive PCR test for influenza within +/− 14 days of the index date (among patients with positive PCR tests for COVID-19), and

*Pr(Flu+ | Co-Tested)*: probability that the individual received a positive PCR test for influenza within +/− 14 days of the index date (among patients with PCR tests available for both influenza and COVID-19).

*Pr(Flu+ | COVID-19+, Co-Tested)*: probability that the individual received a positive PCR test for influenza within +/− 14 days of the index date (among patients with a positive PCR test result for COVID-19 and an influenza PCR test result available).

From the independence assumption, it follows that

$$\begin{equation*}
\Pr \left( {COVID - 19 + } \right) = \Pr \left( {COVID - 19 + |Flu + } \right),and
\end{equation*}
$$

$$\begin{equation*}
\Pr \left( {Flu + } \right) = \Pr \left( {Flu + |COVID - 19 + } \right).
\end{equation*}
$$

In addition, we define the following probabilities of COVID-19 and influenza coinfections:

*Pr(COVID-19+, Flu+)*: probability that the individual received positive PCR tests for both COVID-19 and influenza within +/− 14 days of the index date (among patients with and without PCR testing for COVID-19 and/or influenza),

*Pr(COVID-19+, Flu+* | *Co-Tested)*: probability that the individual received positive PCR tests for both COVID-19 and influenza within +/− 14 days of the index date (among patients with PCR tests available for both influenza and COVID-19).

Similarly, we define the following probabilities that PCR cotesting data is available:

*Pr(Co-Tested)*: probability that the individual received PCR tests for both COVID-19 and influenza within +/− 14 days of the index date,

*Pr(Co-Tested* | *COVID-19+)*: probability that the individual received PCR tests for both COVID-19 and influenza within +/− 14 days of the index date (among patients with a positive PCR test result for COVID-19),

*Pr(Co-Tested* | *Flu+)*: probability that the individual received PCR tests for both COVID-19 and influenza within +/− 14 days of the index date (among patients with a positive PCR test result for influenza), and

*Pr(Co-Tested* | *COVID-19+, Flu+)*: probability that the individual received PCR tests for both COVID-19 and influenza within +/− 14 days of the index date (among patients with a positive PCR test result for both COVID-19 and influenza).

By definition,

$$\begin{equation*}
\Pr \left( {Co - Tested|COVID - 19 + ,Flu + } \right) = 1,
\end{equation*}
$$

because all patients who have received positive PCR tests for both COVID-19 and influenza have received at least 1 PCR test of each type.

Let *n_co-tested_* be the total number of individuals with PCR testing data available for both COVID-19 and influenza within +/− 14 days of the index date. Given these probabilities, we estimate the expected number of coinfection cases at the Mayo Clinic as

$$\begin{eqnarray*}
&& E\left[ {COVID - 19 + ,Flu + |Co - Tested} \right] = {n_{co - tested}}*\Pr ( COVID \nonumber \\&& \quad - \ 19 + ,Flu + |Co - Tested )\nonumber \\&& \quad = \ {n_{co - tested}}*\Pr \left( {COVID - 19 + |Co - Tested} \right)*\Pr ( Flu + |COVID \nonumber \\&&\quad - \ 19 + ,Co - Tested ) \nonumber \\&&\quad = {n_{co - tested}}*\Pr \left( {COVID - 19 + |Co - Tested} \right)\nonumber \\&&\quad * \ \Pr \left( {Flu + |Co - Tested} \right)*c,
\end{eqnarray*}
$$

where

$$\begin{eqnarray*}
c &=& \Pr \left( {Co - Tested} \right)/\left( \Pr \left( {Co - Tested|COVID - 19 + } \right) \right. \nonumber \\&& \left. *{\rm{ }}\Pr \left( {Co - Tested|Flu + } \right) \right).
\end{eqnarray*}
$$

We can interpret this model parameter *c* as the ratio of cotesting PCR rates in the general population relative to the cotesting PCR rates in the COVID-19 and influenza positive populations. Substituting in the actual number of observed COVID-19 and influenza coinfection cases over the study period, we can solve for this model parameter *c* as

$$\begin{equation*}
{c_{estimated}} = \left( {{n_{co - \inf ection}}*{n_{co - tested}}} \right)/\left( {{n_{{\mathop{\rm cov}} id}}*{n_{flu}}} \right),
\end{equation*}
$$

where

*n_coinfection_:* number of coinfected cases,

*n_covid_:* number of cases with positive PCR tests for COVID-19, and

*n_flu_:* number of cases with positive PCR tests for influenza.

In order to estimate the expected number of coinfected cases for a particular time period *t* (e.g. from 2021 December 13), we used the same expected value formula as above with this estimated value for *c*:

$$\begin{eqnarray*}
&& {E_t}\left[ {COVID - 19 + ,{\rm{ }}Flu + {\rm{ }}|{\rm{ }}Co - Tested} \right]{\rm{ }} = \nonumber \\&& \quad {n_{co - tested,{\rm{ }}t}}*{\Pr _t}\left( {COVID - 19 + |Co - Tested} \right)\nonumber \\&& \quad *{\Pr _t}\left( {Flu + |Co - Tested} \right)*{c_{estimated}},
\end{eqnarray*}
$$

where: *n_co-tested, t_, Pr_t_(COVID-19+ | Co-Tested)*, and *Pr_t_(Flu+ | Co-Tested)* are time period-specific case counts and probability estimates.

### Propensity score matching

We used propensity score matching to construct a control cohort of COVID-19 cases without influenza with similar clinical characteristics to the coinfected cohort with at least 30 days of follow-up data. To compute the propensity scores, we trained an L2-regularized logistic regression model with the following covariates defined in the previous sections: demographics (age, sex, race, and ethnicity), geographic location, time period of positive-COVID-19 PCR test, COVID-19 vaccination status, influenza vaccination status, and Elixhauser comorbidities from the past 5 years. Using these propensity scores, we performed 1:1 matching without replacement to find the most similar COVID-19 case in the dataset for each coinfection case. To evaluate the quality of the matching, we computed relative risks with associated 95% CI for each of the matched covariates, which is described in the “Statistical analysis” section. The logistic regression model for propensity score matching was trained using the “sklearn” package (version 1.0.1) in Python.

### Burden of disease assessment based on structured variables and symptomology

For the propensity-matched patients with 30 days of follow-up data available after their COVID-19 diagnosis, we tracked and compared clinical outcomes across the two cohorts. We considered severe outcomes within 30 days including hospitalization, ICU admission, and mortality, which were derived from structured data tables. In addition, we considered the following viral symptoms within 30 days: altered or diminished sense of taste or smell, chest pain/pressure, congestion, conjunctivitis, cough, dermatitis, diaphoresis, diarrhea, dry mouth, fatigue, fever/chills, headache, hemoptysis, myalgia/arthralgia, otitis, pharyngitis, productive cough, respiratory difficulty, rhinitis, and wheezing. These symptoms were extracted from the unstructured clinical notes using the same augmented curation model which was used to determine influenza diagnoses (see “Augmented curation to determine influenza diagnosis from clinical notes”). This list of symptoms was derived from prior research that identified symptoms associated with COVID-19 diagnosis (8). The list of synonyms used to represent each symptom are provided in Table S4 (Supplementary Material).

### Statistical analysis

To identify clinical covariates enriched in the coinfected cohort with respect to the overall COVID-19 cohort, we report relative risk estimates for each of the categorical variables in the dataset, including: demographics, (sex, race, and ethnicity), geographic location and time of PCR test, COVID-19 and influenza vaccination status, and comorbidities. For each categorical covariate, the relative risk was computed as the rate in the coinfected cohort divided by the rate in the control cohort. In addition, we report 95% CI using the Delta method approximation (17). For the numerical covariates, namely age at COVID-19 diagnosis and the Elixhauser comorbidity index, we report summary statistics and *P*-values from the Mann–Whitney *U* test. We repeated these statistical analyses on a subsample of the study population with index dates during the Omicron era (2021 December 14 to 2022 April 2). Similarly, for the propensity-matched cohorts, we reported relative risks and associated 95% CI for each of the 30-day outcome variables, including: hospitalization, ICU admission, mortality, and viral symptoms. Relative risks, 95% CI, and *P*-values from the Mann–Whitney *U* test were computed using the “scipy” package (version 1.7.2) in Python.

### Data and code availability statement

The data, associated protocols, code, and materials for this study may be made available from the corresponding author on request. A proposal with detailed description of study objectives and statistical analysis plan will be needed for evaluation of the reasonability of requests. Deidentified data will be provided after approval from the lead contact and the Mayo Clinic's standard IRB process for such requests.

## Results

### Recent surges in COVID-19 and influenza cases correspond with recent rises in the number of hospitalized patients with COVID-19 infection, influenza infection, and coinfections in the United States

**Fig. 1.**
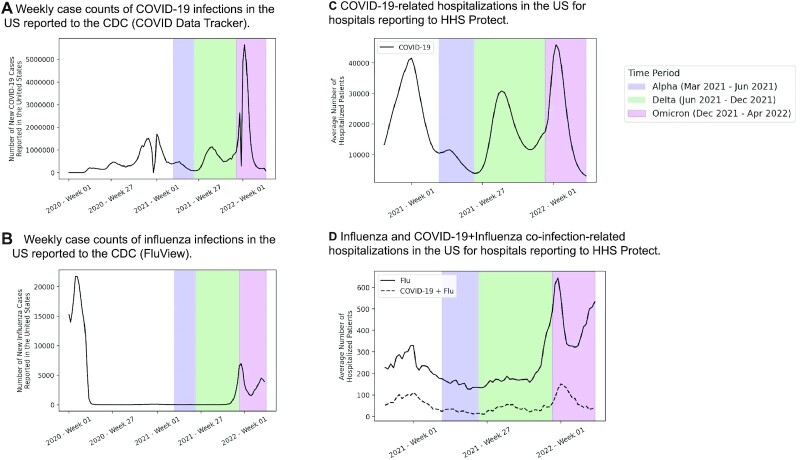
Case counts and hospitalizations for COVID-19, influenza, and coinfections in the United States. (A) Weekly case counts of new COVID-19 infections in the United States reported by the CDC COVID Data Tracker. (B) Weekly case counts of new influenza infections in the United States reported by CDC FluView. (C) Counts of COVID-19 hospitalized cases. (D) Counts of influenza and coinfection hospitalized cases. (A)–(D) In each of the plots, periods of time corresponding to the different waves of the pandemic are shaded, including Alpha (2021 March 16 to 2021 June 15; blue), Delta (2021 June 16 to 2021 December 13; green), and Omicron (2021 December 14 to 2022 April 2; purple).

In Fig. 1, we show trends in COVID-19 and influenza infections, hospitalizations, and coinfection-related hospitalizations across the United States, aggregated from the CDC COVID Data Tracker, CDC FluView, and HHS Protect Public Data Hub. COVID-19 infection rates have risen to all time highs during the Omicron era (Fig. 1A), while influenza infection rates have been very low throughout the pandemic until recently at the beginning of the 2021 to 2022 flu season (Fig. 1B). Although we are unable to determine coinfection rates in the overall US population, we can do so for hospitalized patients from HHS Protect data. In Fig. 1(C) and (D), we show the average number of hospitalized patients with COVID-19 infection, influenza infection, and coinfections for 3,003 hospitals with data reporting to HHS Protect. We observe large peaks in hospitalized COVID-19 patients during the original (Wuhan strain), Delta, and Omicron waves of the pandemic (Fig. 1C). On the other hand, hospitalized influenza case counts were slightly elevated during the 2020 to 2021 flu season, but have dramatically increased starting in October 2021 (Fig. 1D). Case counts of hospitalized coinfected patients follow a similar trend, with a slight elevation during the 2020 to 2021 flu season and rising case counts observed from December 2021 to April 2022 (Fig. 1D).

### The recent rise in hospitalized coinfection cases corresponds with the Omicron SARS-CoV-2 and H3N2 influenza strains

**Fig. 2.**
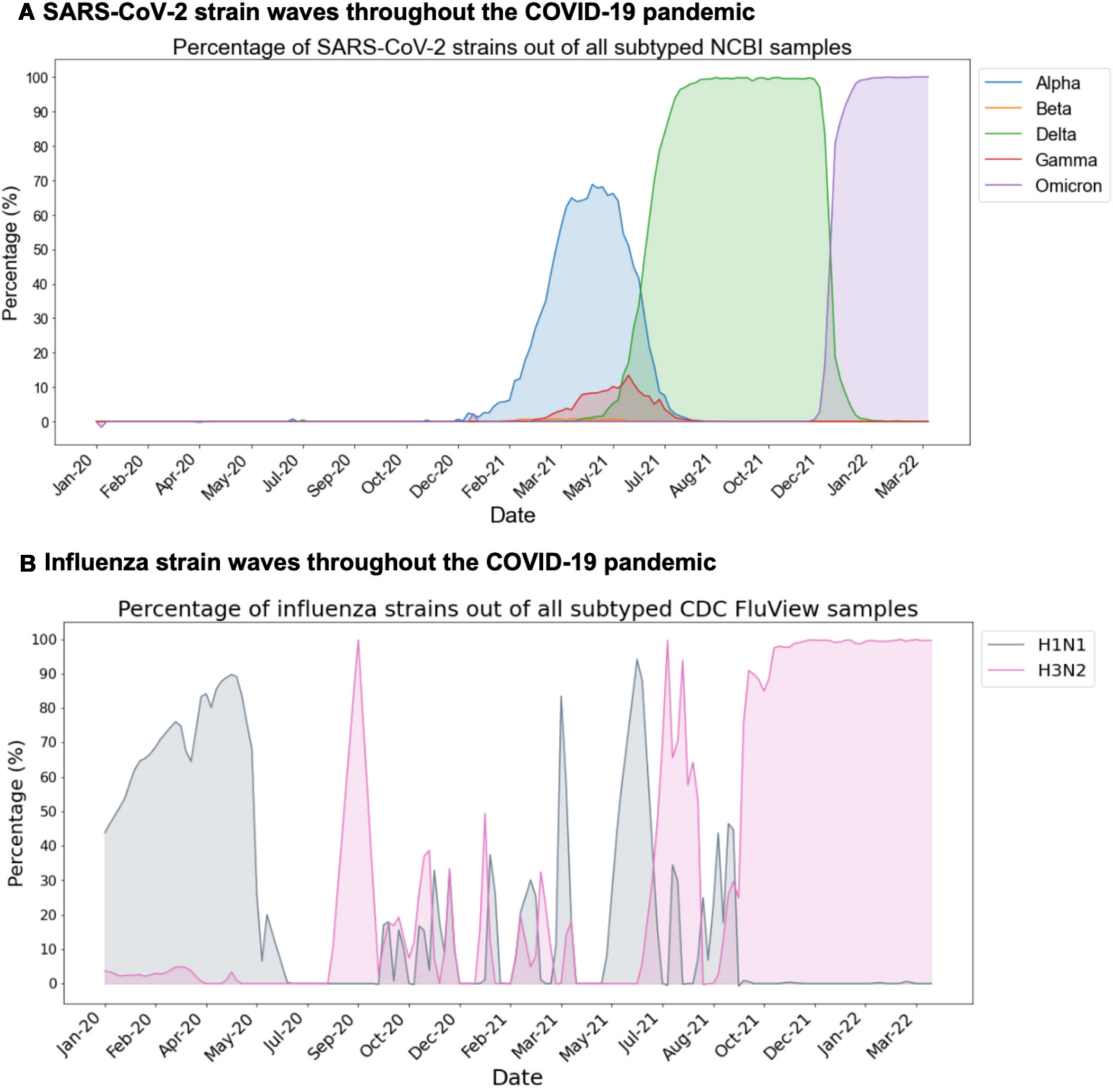
Trends of SARS-CoV-2 and influenza strain prevalences in the United States over time. (A) SARS-CoV-2 strain percentages in the United States are shown from the NCBI data, including the following strains: Alpha (blue), Beta (orange), Delta (green), Gamma (red), and Omicron (purple). In this plot, prevalence of the original (Wuhan) strain is not shown. (B) Influenza strain percentages in the United States are shown from the CDC FluView data, including the following strains: A(H1N1)pdm09 (gray) and A(H3N2) (pink). In both panels (A) and (B), less common strains are omitted from the plots so the strain percentages do not necessarily add up to 100% each day.

In the plots for Fig. 1, we highlight the time periods corresponding to different waves of the COVID-19 pandemic, including time periods when the Alpha, Delta, and Omicron variants were the most prevalent SARS-CoV-2 strains. Next, we provide a more granular view of the SARS-CoV-2 and influenza strains estimated to be in circulation in the United States over the course of the pandemic. In Fig. 2, we provide the estimated percentages of individual SARS-CoV-2 and influenza strains sequenced in the United States based on the NCBI Virus and CDC FluView databases, with SARS-CoV-2 strains including Alpha, Beta, Delta, Gamma, and Omicron, and influenza strains including H1N1 and H3N2. Taken in combination with the HHS Protect data, the recent rise in coinfection-related hospitalizations has occurred while the Omicron SARS-CoV-2 variant and the H3N2 influenza strain were most prevalent in the United States.

### The recent surge in coinfection cases observed at the Mayo Clinic coincides with recent surges in both COVID-19 and influenza cases observed at the health system

**Fig. 3.**
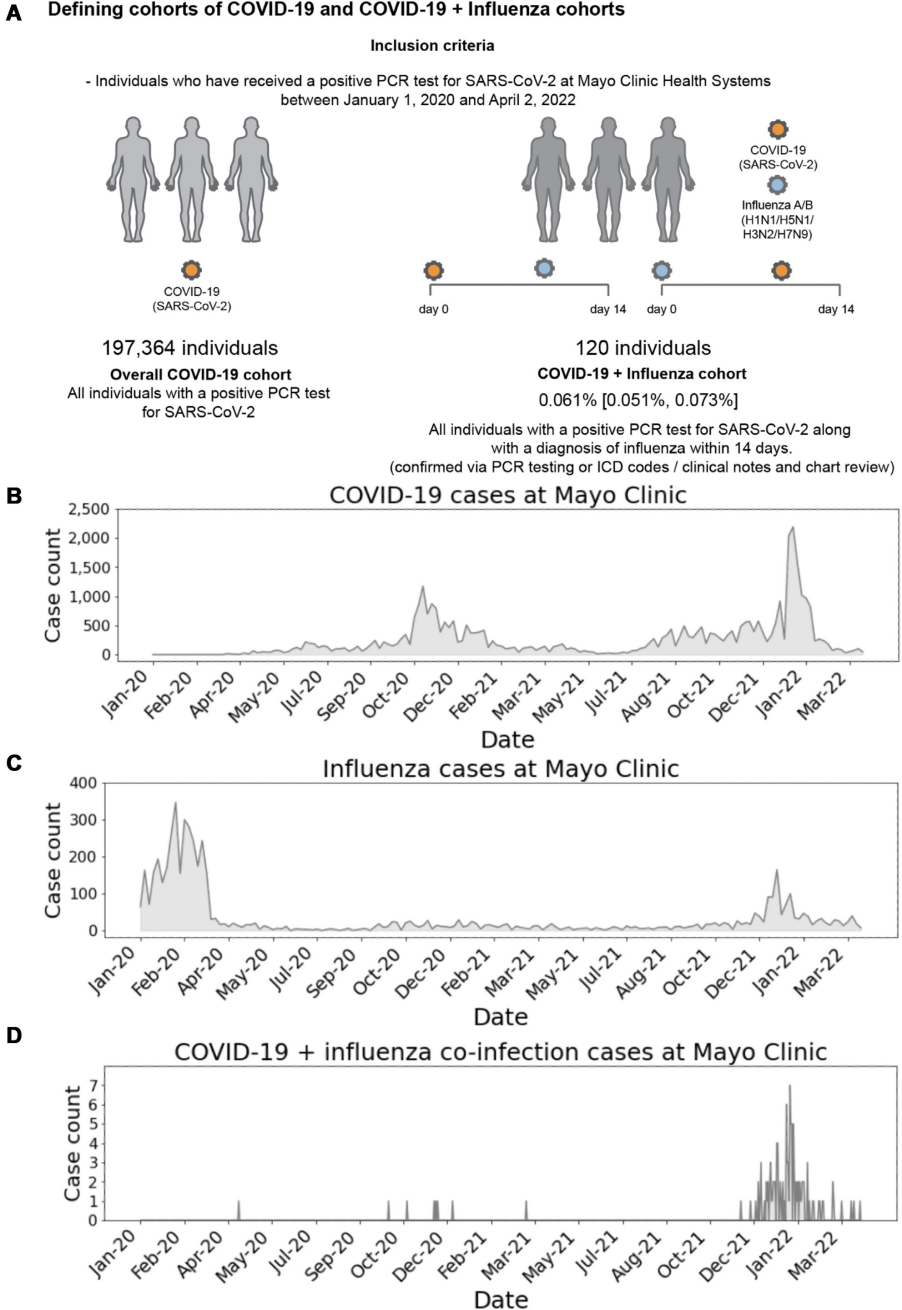
Schematic representation of cohort definitions and analysis of daily case counts of COVID-19 infection, influenza infection, and coinfections at the Mayo Clinic. (A) The inclusion criteria of COVID-19 and coinfection cohorts based on the Mayo Clinic EHR data and the resulting cohort sizes are shown. Case counts of COVID-19 (B) and influenza (C). (D) Case counts of COVID-19 and influenza coinfection cases including 120 cases determined via PCR testing, ICD codes, and/or clinical notes. (B)–(D) The *y*-axis ranges in each plot are different, and the case counts for COVID-19 infection are significantly greater than the case counts for influenza infection which are in turn significantly greater than the case counts for COVID-19 and influenza coinfection.

EHR data from Mayo Clinic corroborates the observed trend in the public data that coinfection cases are increasing along with rises in COVID-19 and influenza (Fig. 3). Coinfection cases were defined based on features in the EHR data as described in the Methods and summarized in Fig. 3(A). New cases of COVID-19 and influenza at the Mayo Clinic (Fig. 3B and C) follow similar trends to those seen on the national level (Fig. 1A and B). In Table S5 (Supplementary Material), we provide case counts of COVID-19 infections, influenza infections, and coinfections observed at the Mayo Clinic during each of the four study time periods (corresponding to the Wuhan (original), Alpha, Delta, and Omicron waves).

### Laboratory testing for COVID-19 and influenza coinfections is low and confirmed coinfections are rare

Among 197,364 COVID-19 cases at the Mayo Clinic, only 17,932 (9.1%) cases had an influenza PCR test recorded within 2 weeks of their positive SARS-CoV-2 PCR test (Table 1). Of these 17,932 cotested cases, only 103 individuals tested positive for influenza, resulting in an estimated lab-confirmed coinfection prevalence of 0.574% (95% CI: [0.474%, 0.696%]). Given the possibility of incomplete lab data in the EHR, we also considered other documentation methods of influenza diagnosis, which increased the size of the coinfected cohort to 120 cases (four additional cases determined from ICD codes and 13 additional cases determined from augmented curation of clinical notes). The notes associated with the 13 additional cases found by augmented curation of the clinical notes were manually reviewed. In most cases, it was found that the positive influenza test occurred outside of the Mayo Clinic and the information was retroactively entered into the patient chart. Otherwise, the influenza diagnosis was referenced in at least one note but the source of diagnosis was unspecified. When we consider these additional data modalities for influenza diagnosis, the pool of eligible coinfection cases is the overall COVID-19 population (*n* = 197,364). In this eligible population, the prevalence estimate of influenza coinfection from all three of these data sources is 0.061% (95% CI: [0.051%, 0.073%]; Table 1). Among the population with PCR-confirmed influenza during the study period (*n* = 2,919), the prevalence of COVID-19 coinfection was 3.53% [2.92%, 4.26%] (Table 1).

**Table 1.** Case counts and estimated prevalences for COVID-19 and influenza coinfections from the Mayo Clinic EHR data.

(a) Case counts of coinfections from COVID-19 cohort.		
Cohort	Case count (%)	
All COVID-19 cases (confirmed by PCR test)	197,364 (100.0%)	
COVID-19 cases with lab tests for influenza within +/− 14 days	17,932 (9.1%)	
COVID-19 and influenza coinfection within +/− 14 days, with diagnosis of influenza confirmed by:		
- Lab test	103 (0.05%)	
- ICD-10 codes + manual review	4 (0.00%)	
- Clinical notes + manual review	13 (0.01%)	
- Any of the above	120 (0.06%)	
(b) Case counts of coinfections from influenza cohort.		
**Cohort**	**Case count (%)**	
All influenza cases during the study period (2020 January 1 to 2022 April 2)	2,919 (100%)	
COVID-19 and influenza coinfection within +/− 14 days, with diagnosis of influenza confirmed by:		
- Lab test	103 (3.5%)	
c) Prevalence estimates.		
**Description**	**Cases/population**	**Prevalence (95% CI)**
Estimated prevalence of COVID-19 and influenza coinfections from overall COVID-19 cohort	120/197,364	0.061% [0.051%, 0.073%]
Estimated prevalence of COVID-19 and influenza coinfections from COVID-19 cohort with lab tests available	103/17,932	0.574% [0.474%, 0.696%]
Estimated prevalence of COVID-19 and influenza coinfections from overall Influenza cohort	103/2,919	3.53% [2.92%, 4.26%]

### Coinfection with SARS-CoV-2 and influenza is not more frequent than expected by chance

We next asked whether coinfections have occurred at unexpectedly high rates during various intervals throughout the pandemic, given the background rates of COVID-19 and influenza. For each time period analyzed, the observed number of coinfection cases is equal to or slightly below the expected number of coinfection cases (Table 2). In particular, during the Omicron era (from 2021 December 14 to 2022 April 2), the expected number of COVID-19 and influenza coinfection cases among this population was 107.5 cases (95% CI: [102.9,112.0]), while the observed count was only 94 cases. During the Delta era (from 2021 June 16 to 2021 December), the expected number of coinfection cases in this population was 13.6 (95% CI: [12.4, 14.8]), but only nine coinfection cases were observed. These results suggest that after controlling for the background rates of both COVID-19 and influenza, the number of coinfections is not higher than expected by chance.

**Table 2.** Expected number of coinfections throughout the pandemic among patients at the Mayo Clinic with cotesting PCR data. In the first column, we show the total number of cases at the Mayo Clinic during the time period with PCR testing data available for both COVID-19 and influenza within +/− 14 days, including both positive and negative PCR tests. For each row, this total is used as the denominator to compute the percentages, which are displayed next to case counts. In the middle columns, we show the number of cases with positive PCR tests for COVID-19, influenza, and both COVID-19 and influenza, respectively. In the last column, we show the expected number of cases with positive PCR tests for both COVID-19 and influenza, assuming that the probabilities of testing positive for COVID-19 and influenza are independent in the general population (see Methods). Note that for the first two time periods, all confirmed coinfection cases had influenza diagnoses determined via ICD codes or clinical notes and not lab tests, so the coinfection case counts for these time periods were zero.

Time period	Cases with PCR testing for both COVID-19 and Flu within 14 days	COVID-19 positive tests case count (%)	Flu positive tests case count (%)	COVID-19 + Flu positive tests case count (%)	Expected number of COVID-19 + Flu positive tests case count (%) [95% CI]
2020 March 12 to 2021 March 15	14,320	1,162 (8.1%)	165 (1.2%)	0 (0.0%)	2.6 (0.02%)
[2.2 (0.02%), 3.0 (0.02%)]					
2021 March 16 to 2021 June 15	1,806	42 (2.3%)	0 (0.0%)	0 (0.0%)	0.0 (0.0%)
[0.0 (0.0%), 0.0 (0.0%)]					
2021 June 16 to 2021 December 13	28,946	3,918 (13.5%)	520 (1.8%)	9 (0.0%)	13.6 (0.05%)
[12.4 (0.04%), 14.8 (0.05%)]					
2021 December 14 to 2022 April 2	57,488	13,740 (23.9%)	2,332 (4.1%)	94 (0.2%)	107.5 (0.19%)
[102.9 (0.18%), 112.0 (0.19%)]					

### COVID-19 and influenza coinfections are associated with time and location of PCR test, reinfections, COVID-19 vaccine status, COVID-19 vaccine type, race/ethnicity, and age

We next compared the clinical characteristics of the overall COVID-19 cohort (*n* = 197,364 cases) to the clinical characteristics of the coinfected cohort (*n* = 120 cases; Table 3). The coinfected cohort was significantly younger on average, with a mean age of 26.7 years old (SD: 20.7 years) compared to 49.0 years old (SD: 20.7 years) for the overall COVID-19 cohort. Other clinical covariates such as ethnicity and pre-existing conditions were similar between the two cohorts (Table 3). As expected, most coinfection cases (*n* = 102, 85.0%) occurred during the Omicron era (2021 December 14 to 2022 April 2). Compared to the overall COVID-19 cohort, coinfection cases were more likely to be COVID-19 reinfections (RR: 2.31, 95% CI: [1.31, 4.45]), to occur in individuals who were fully vaccinated (RR: 1.61, 95% CI: [1.20, 2.19]), and to occur in individuals vaccinated with the Moderna COVID-19 vaccine (RR: 2.23, 95% CI: [1.47, 3.51]; Table 3). We note that each of these covariates (reinfections, full vaccination, and Moderna vaccination) are more prevalent later in the pandemic, which could explain their positive associations with coinfection. We also observed that coinfection cases were slightly higher in the Mayo Clinic—Midwest region compared to the other regions (RR: 1.17, 95% CI: [1.11, 1.22]) and slightly higher in certain racial and ethnic subgroups (race—Native American RR: 3.62, 95% CI: [1.31, 15.33]; race—other RR: 1.91, 95% CI: [1.05, 3.85]; and ethnicity—unknown RR: 0.13, 95% CI: [0.04, 0.97]).

**Table 3.** Clinical characteristics of “COVID-19 + Flu” and “Overall COVID-19” cohorts at the Mayo Clinic. The “COVID-19 + Flu” cohort includes all individuals with a positive PCR test for SARS-CoV-2 and at least one of the following within 14 days: a positive laboratory test for influenza, an ICD code for influenza, or a clinical note indicating a diagnosis of influenza. The “Overall COVID-19” cohort includes all individuals with a positive PCR test for SARS-CoV-2. ^*** ^implies significant value.

Clinical characteristic	COVID-19 + Flu cohort case count (%)	Overall COVID-19 cohort case count (%)	Relative risk (95% CI)
Total number of cases	120	197,364	
Type of SARS-CoV-2 infection			
- Primary infection	111 (92.5%)	190,916 (96.7%)	0.96 [0.90, 1.00]
- Reinfection	9 (7.5%)	6,448 (3.3%)	2.31 [1.31, 4.45]***
Time of SARS-CoV-2 infection			
- 2020 March 12 to 2021 March 15	7 (5.8%)	77,989 (39.5%)	0.15 [0.08, 0.31]***
- 2021 March 16 to 2021 June 15	1 (0.8%)	7,685 (3.9%)	0.21 [0.06, 1.56]
- 2021 June 16 to 2021 December 13	10 (8.3%)	49,705 (25.2%)	0.33 [0.19, 0.61]***
- 2021 December 14 to 2022 April 2	102 (85.0%)	61,952 (31.4%)	2.71 [2.50, 2.91]***
COVID-19 vaccination status			
- Unvaccinated	83 (69.2%)	150,102 (76.0%)	0.91 [0.81, 1.02]
- Partial	6 (5.0%)	5,843 (3.0%)	1.69 [0.86, 3.83]
- Full	31 (25.8%)	31,624 (16.0%)	1.61 [1.20, 2.19]***
- Boosted	0 (0.0%)	9,795 (5.0%)	0.00 [0.00, 1.32]
Initial COVID-19 vaccine type			
- None	83 (69.2%)	150,102 (76.0%)	0.91 [0.81, 1.02]
- Janssen	3 (2.5%)	3,065 (1.6%)	1.61 [0.66, 5.23]
- Moderna	17 (14.2%)	12,547 (6.4%)	2.23 [1.47, 3.51]***
- Pfizer/BioNTech	17 (14.2%)	31,650 (16.0%)	0.88 [0.58, 1.39]
Flu vaccination status at time of SARS-CoV-2 infection			
- Unvaccinated	104 (86.7%)	175,327 (88.9%)	0.98 [0.91, 1.04]
- Vaccinated	16 (13.3%)	21,971 (11.1%)	1.20 [0.78, 1.92]
Site			
Mayo Clinic—Arizona	3 (2.5%)	18,910 (9.6%)	0.26 [0.11, 0.85]***
Mayo Clinic—Florida	5 (4.2%)	20,686 (10.5%)	0.40 [0.19, 0.98]***
Mayo Clinic—Midwest	112 (93.3%)	157,768 (79.9%)	1.17 [1.11, 1.22]***
Sex			
- Female	53 (44.2%)	101,039 (51.2%)	0.86 [0.71, 1.06]
- Male	67 (55.8%)	96,254 (48.8%)	1.14 [0.98, 1.34]
- Unknown/nonbinary	0 (0.0%)	71 (0.0%)	0.00 [0.00, 183.02]
Race			
- Asian	2 (1.7%)	5,157 (2.6%)	0.23, 2.70]
- Black/African American	8 (6.7%)	8,438 (4.3%)	0.86, 3.15]
- Native American	2 (1.7%)	912 (0.5%)	1.31, 15.33]***
- Native Hawaiian/Pacific Islander	0 (0.0%)	354 (0.2%)	0.00, 36.63]
- White/Caucasian	98 (81.7%)	165,701 (84.0%)	[0.89, 1.06]
- Other	8 (6.7%)	6,883 (3.5%)	1.05, 3.85]***
- Unknown	2 (1.7%)	9,919 (5.0%)	0.33 [0.12, 1.40]
Ethnicity			
- Hispanic or Latino	11 (9.2%)	14,660 (7.4%)	0.74, 2.22]
- Not Hispanic or Latino	108 (90.0%)	170,335 (86.3%)	1.04 [0.98, 1.10]
- Unknown	1 (0.8%)	12,369 (6.3%)	0.13 [0.04, 0.97]***
Clinical characteristic	COVID-19 + Flu cohort case count (%)	Overall COVID-19 cohort case count (%)	Mann–Whitney *U* test *P*-value
Age at time of positive PCR test for SARS-CoV-2 (in years)			
- Mean:	26.7	49.0	3.2e-12***
- Median:	19.9	38.7	
- SD:	20.7	21.2	
- IQR:	(11.6, 40.8)	(23.1, 56.4)	
Elixhauser Comorbidity Index			
- Mean:	0.7	1.7	0.23
- SD:	5.4	6.5	

Restricting the analysis to the Omicron era (2021 December 14 to 2022 April 2) to account for time period as a potential confounding factor, some enrichments in the coinfected cohort are no longer statistically significant (Table S6, Supplementary Material). In this subpopulation, coinfection cases were more likely to occur in individuals who were unvaccinated (RR: 1.28, 95% CI: [1.11, 1.47]), located in the Mayo Clinic—Midwest region (RR: 1.29, 95% CI: [1.24, 1.34]), in a particular racial subgroup (race—Native American RR: 4.34, 95% CI: [1.57, 18.34]; race—other RR: 2.17, 95% CI: [1.16, 4.60]), and were younger in age (Mann–Whitney *U* test *P*-value: < 0.001; Table S6, Supplementary Material). In particular, during the Omicron era, coinfection rates were not elevated among fully vaccinated individuals or among individuals who received the Moderna vaccine. Notably, there were no coinfection cases during this time period among individuals who have received COVID-19 booster vaccine doses (RR: 0.00, 95% CI: [0.00, 0.52]). In addition, there was a lower coinfection rate among individuals who received the Pfizer/BioNTech COVID-19 vaccine (RR: 0.47, 95% CI: [0.31, 0.75]) compared to individuals who received another COVID-19 vaccine type or were unvaccinated.

### Viral symptoms are more likely to occur in coinfected patients

Among the 120 coinfection cases at Mayo Clinic, 115 patients had at least 30 days of follow-up after their positive SARS-CoV-2 PCR test. These 115 coinfected patients were matched to COVID-19 monoinfection patients based on demographics, clinical characteristics, vaccination status, and time of COVID-19 diagnosis via propensity score matching (Table S7, Supplementary Material). In the 30 day follow-up period, there were no significant differences in severe clinical outcomes between the coinfected cohort and the propensity-matched COVID-19 mono-infection cohort (1 vs. 0 hospitalizations, 1 vs. 0 ICU admissions, and 0 vs. 0 deaths; Table 4). Several viral symptoms were reported at higher rates in the coinfected cohort than in the matched COVID-19 monoinfection cohort. The complications that were more common among patients in the coinfected cohort included congestion (RR: 3.80, 95% CI: [1.43, 8.81]), cough (RR: 3.10, 95% CI: [1.57, 5.74]), fever/chills (RR: 2.80, 95% CI: [1.40, 5.25]), headache (RR: 4.20, 95% CI: [1.59, 9.62]), myalgia/arthralgia (RR: 4.20, 95% CI: [1.59, 9.62]), pharyngitis (RR: 3.00, 95% CI: [1.10, 7.20]), and rhinitis (RR: 4.33, 95% CI: [1.22, 12.15]; Table 4).

**Table 4.** Outcomes of matched “COVID-19 + Flu” and “COVID-19 monoinfection” cohorts at the Mayo Clinic. The first cohort “Matched COVID-19 + Flu” includes all individuals with a confirmed coinfection of SARS-CoV-2 and influenza within 14 days along with at least 30 days of follow-up data. The second cohort “Matched COVID-19 monoinfection” includes all individuals who were selected as matched controls for the first cohort via propensity score matching with exact matching on the time period of infection (see Methods). Eligible matches for the second cohort include all individuals with a confirmed SARS-CoV-2 monoinfection (i.e. no coinfection with influenza) with at least 30 days of follow-up data. *** implies significant value.

impOutcome	Matched COVID-19 + Flu cohort case count (%)	Matched COVID-19 monoinfection cohort case count (%)	Relative risk (95% CI)
Total number of cases	115	115	
Outcomes from structured data (within 30 days of COVID-19 diagnosis)			
- Hospitalization	1 (0.8%)	0 (0.0%)	Undefined
- ICU admission	1 (0.8%)	0 (0.0%)	Undefined
- Death	0 (0.0%)	0 (0.0%)	NA
Outcomes from clinical notes (within 30 days of COVID-19 diagnosis)			
- Altered/diminished sense of taste or smell	2 (1.7%)	0 (0.0%)	Undefined
- Chest pain/pressure	6 (5.2%)	3 (2.6%)	0.52, 6.64]
- Congestion	19 (16.5%)	5 (4.3%)	1.43, 8.81]***
- Conjunctivitis	0 (0.0%)	0 (0.0%)	NA
- Cough	31 (27.0%)	10 (8.7%)	1.57, 5.74]***
- Dermatitis	3 (2.6%)	2 (1.7%)	1.50 [0.28, 6.95]
- Diaphoresis	4 (3.5%)	1 (0.9%)	4.00 [0.48, 18.70]
- Diarrhea	9 (7.8%)	6 (5.2%)	1.50 [0.56, 3.83]
- Dry mouth	0 (0.0%)	0 (0.0%)	NA
- Fatigue	15 (13.0%)	8 (7.0%)	1.88 [0.82, 4.04]
- Fever/chills	28 (24.3%)	10 (8.7%)	2.80 [1.40, 5.25]***
- Headache	21 (18.3%)	5 (4.3%)	4.20 [1.59, 9.62]***
- Hemoptysis	0 (0.0%)	0 (0.0%)	NA
- Myalgia/arthralgia	21 (18.3%)	5 (4.3%)	4.20 [1.59, 9.62]***
- Otitis	1 (0.9%)	1 (0.9%)	1.00 [0.11, 9.47]
- Pharyngitis	15 (13.0%)	5 (4.3%)	3.00 [1.10, 7.20]***
- Productive cough	4 (3.5%)	0 (0.0%)	Undefined
Respiratory difficulty	12 (10.4%)	9 (7.8%)	0.59, 2.94]
- Rhinitis	13 (11.3%)	3 (2.6%)	4.33 [1.22, 12.15]***
- Wheezing	3 (2.6%)	3 (2.6%)	1.00 [0.23, 4.30]

## Discussion

Since the COVID-19 pandemic began in early 2020, cases of influenza have been dwarfed by cases of COVID-19. Several studies have shown that rates of influenza were lower in the 2020 to 2021 flu season, which has been largely attributed to social distancing measures for COVID-19 (18–20). In our study population with COVID-19, the rate of lab testing for influenza coinfections was very low (9.1%). We expect that the low number of cases during the 2020 to 2021 flu season may explain why few influenza tests were ordered during the study time period, since most individuals would have a low pretest probability for flu and a high pretest probability for COVID-19. Starting in November 2021, the Mayo Clinic Rochester, MN site started to systematically test for COVID-19 when testing for influenza, but before this, testing for COVID-19 and influenza was entirely dependent on provider suspicion. Even among individuals with both SARS-CoV-2 PCR testing and influenza PCR testing data available, we found that coinfection cases were rare, with an estimated prevalence of 0.574% (95% CI: [0.474%, 0.696%]) based upon a sample of 17,932 cotested individuals. This is in line with previously estimated coinfection prevalences of 0.8% worldwide and 0.4% in the United States (3).

Compared to the overall COVID-19 study population, the cohort of coinfected individuals at the Mayo Clinic had higher rates of several clinical covariates including: coinfection during the Omicron era (2021 December 14 to 2022 April 2), geographic location (Mayo Clinic—Midwest), SARS-CoV-2 reinfection, COVID-19 vaccination status (full), COVID-19 vaccine type (Moderna), younger age, race (Native American and other), and ethnicity (unknown). Since the vast majority of coinfection cases were observed during the Omicron era, any features associated with later time periods would also be associated with higher rates of coinfection. This explains why covariates such as vaccination status, reinfection, and vaccine type are all associated with higher rates of coinfection. Indeed, a subsample analysis of cases during the Omicron era showed that coinfected cases during this time period did not have higher rates of SARS-CoV-2 reinfection, COVID-19 vaccination status (full), or COVID-19 vaccine type (Moderna), which supports this hypothesis. In addition, differences in coinfection rates across the Mayo Clinic sites could be explained by differences in cotesting rates at these sites.

Furthermore, in this study we show that the elevated coinfection case counts observed during the Omicron era are in line with the expected numbers of coinfected cases given the background prevalences of COVID-19 and influenza in the Mayo Clinic population. From the epidemiological analysis of the HHS Protect data, rates of coinfection-related hospitalizations have tracked closely with the rates of influenza-related hospitalizations in the United States. Together, this data suggests that recently observed increased rates of COVID-19 and influenza coinfections in the United States are most likely directly attributable to the recent surge in both COVID-19 and influenza cases during the 2021 to 2022 flu season rather than other factors such as the emergence of the Omicron variant. Aside from the time of SARS-CoV-2 infection, other factors may influence the risk of coinfection which could explain the enrichments among the coinfected cohort. For example, young males may be less likely to adhere to social distancing interventions, which would explain the higher prevalence of coinfections in this group. In addition, the higher rates of coinfections observed in the Mayo Clinic—Midwest region may be due to testing differences between the sites.

In addition to assessing the baseline characteristics of coinfected cases, we also evaluated their 30-day clinical outcomes. Among the 115 coinfected cases with 30-day follow-up data available, we observed one hospital/ICU admission and no deaths. Similarly, we observed no cases of hospital/ICU admission and no deaths among the 1:1 propensity matched cohort. These results provide preliminary evidence that severe outcomes are no more likely to occur for coinfected patients compared to monoinfected COVID-19 patients with similar baseline characteristics. On the other hand, symptoms associated with viral infections including congestion, cough, fever/chills, headache, myalgia/arthralgia, pharyngitis, and rhinitis were elevated in the coinfected population relative to the propensity-matched COVID-19 monoinfection cohort. This may be due to differences in background testing rates since asymptomatic individuals were more likely to undergo routine screening for SARS-CoV-2 monoinfection rather than SARS-CoV-2 and influenza coinfection during the study period. Going forward, it will be important to monitor the clinical outcomes of COVID-19 and influenza coinfection cases among a larger population of individuals with risk factors including older age, obesity, and immunocompromised status.

There are several important limitations to note for this study. First, this epidemiological analysis only includes data from US hospitals, which have reported coinfections to the HHS Protect Public Data Hub. As a result, this dataset does not include all hospitals. Second, laboratory testing rates for influenza coinfections among COVID-19 cases are low, so the CI for the prevalence of COVID-19 and influenza coinfections based on laboratory data alone is large. Third, the criteria for COVID-19 diagnosis used in this study is a positive PCR test for SARS-CoV-2, which includes both symptomatic and asymptomatic COVID-19 cases. We may have obtained different results if we had used a different definition for COVID-19 diagnosis. For example, if we restricted the definition to symptomatic positive PCR tests for SARS-CoV-2, then the overall rates of symptoms in the coinfection and propensity-matched cohorts would have been higher and the differences in symptom profiles may have been less pronounced. Further, COVID-19 was not diagnosed through augmented curation of the clinical notes or through ICD codes with subsequent manual chart review, as was done for influenza cases. As a result, it is possible that we missed COVID-19 cases that were diagnosed by lab testing outside of the Mayo Clinic (including at-home rapid tests) and subsequently recorded in structured diagnosis codes or the clinical notes. Fourth, data on COVID-19 vaccination status for this study population may be incomplete. For example, some individuals who were labeled as “unvaccinated” may have received vaccines for COVID-19 outside of the Mayo Clinic Health Systems that were not linked state vaccine registries, which would not be recorded in the EHR. Fifth, in the probability model to estimate the expected number of coinfection cases at the Mayo Clinic, we assume that probabilities of COVID-19 infection and influenza infection are independent, but the true underlying probability distributions are most likely more complex. Indeed, given that the low number of influenza cases throughout most of 2020 to 2021 has been attributed largely to the nonpharmaceutical interventions used to curb COVID-19 (21), and compliance with these measures varied according to COVID-19 prevalence, there certainly is a complex interaction that cannot be fully modeled here. This is further complicated in the present flu season with reimplementation of nonpharmaceutical interventions in several localities during the present Omicron surge. Sixth, while we control for pre-existing conditions in the propensity-matched analysis, currently we are not controlling for other factors such as respiratory symptoms at time of presentation, which may impact testing rates for influenza and COVID-19 or the availability of a combined test. Finally, the EHR dataset only includes data on COVID-19 cases from a single healthcare system in the United States, which serves a patient population with a unique set of demographic and clinical characteristics in specific geographic areas of the United States (the Midwest, Arizona, and Florida). To assess prevalence and clinical outcomes for COVID-19 and influenza coinfections in the broader population, similar studies in other healthcare systems will be required.

Taken together, these data suggest that COVID-19 and influenza coinfection occurs infrequently, and thus far coinfection rates during the Omicron era are no higher than expected given the background prevalences of both COVID-19 and influenza. Hospitalization and mortality with the combined illness appears to be very rare, possibly attributed to the observed cases skewing younger than the overall COVID-19 population. All viral symptoms are more common among coinfection cases of COVID-19 and influenza compared to monoinfection cases of COVID-19, which are presumably attributable to the influenza secondary diagnoses. Characterization of the symptoms typically associated with coinfection cases could lead to more targeted testing, earlier diagnosis for concurrent viral infections, and better patient outcomes. As SARS-CoV-2 and influenza continue to circulate in the United States and abroad, continued surveillance of coinfection cases will be important especially among subpopulations at high risk for severe disease from viral infections.

## Funding

A.D.B. is supported by grants from the NIAID (grants AI110173 and AI120698), Amfar (#109593), and the Mayo Clinic (HH Shieck Khalifa Bib Zayed Al-Nahyan Named Professorship of Infectious Diseases).

## Authors’ Contributions

CP and VS designed the study. All authors conducted the study and wrote the paper.

## Supplementary Material

pgac071_Supplemental_File

## Notes

**Competing Interest:** C.P., E.S., P.J.L., E.G., K.M., J.R., A.J.V., and V.S. are employees of nference and have financial interests in the company. Mayo Clinic holds equity in nference as a strategic investor and has financial interests in the company. nference is collaborating with biopharmaceutical companies on data science initiatives unrelated to this study. These collaborations had no role in the study design, data collection and analysis, decision to publish, or preparation of the manuscript. J.C.O. has received small grants from nference, Inc., and personal consulting fees from Bates College and Elsevier Inc. All of these activities are outside of the present work. A.D.B. is a paid consultant for Abbvie, Gilead, Freedom Tunnel, Pinetree therapeutics Primmune, Immunome, MarPam, Rion, Symbiosis NexImmune, and Flambeau Diagnostics, is a paid member of the DSMB for Corvus Pharmaceuticals, Equilium, CSL Behring, and Excision Biotherapeutics, has received fees for speaking for Reach MD, Peer Voice, and Medscape, owns equity for scientific advisory work in Zentalis Rion and Nference, and is the founder and President of Splissen therapeutics, and the Member of the Board of Attivare. D.W.C., H.L.G., and L.L.S. have no interests to disclose. This research is being conducted in compliance with the Mayo Clinic conflict of interest policies.
