# Supplementary material for: SARS-CoV-2 and influenza coinfection throughout the COVID-19 pandemic: an assessment of coinfection rates, cohort characteristics, and clinical outcomes
Source: PNAS Nexus. 2022 Jul 4;1(3):pgac071. doi: 10.1093/pnasnexus/pgac071 (PMC9291226; doi:10.1093/pnasnexus/pgac071)
Supplement: pgac071_Supplemental_File [file pgac071_supplemental_file.docx]

**Supplementary Materials**

**Table S1: LOINC codes associated with influenza lab tests.**

| **LOINC code** | **Description** | **Influenza Type/Subtype** |
| --- | --- | --- |
| 34487-9 | Influenza virus A RNA [Presence] in Specimen by NAA with probe detection | Influenza A |
| 40982-1 | Influenza virus B RNA [Presence] in Specimen by NAA with probe detection | Influenza B |
| 49521-8 | Influenza virus A H1 RNA [Presence] in Specimen by NAA with probe detection | Influenza A/H1N1 |
| 49524-2 | Influenza virus A H3 RNA [Presence] in Specimen by NAA with probe detection | Influenza A/H3N2 |
| 76077-7 | Influenza virus A RNA [Presence] in Bronchoalveolar lavage by NAA with probe detection | Influenza A |
| 76078-5 | Influenza virus A RNA [Presence] in Nasopharynx by NAA with probe detection | Influenza A |
| 76079-3 | Influenza virus B RNA [Presence] in Bronchoalveolar lavage by NAA with probe detection | Influenza B |
| 76080-1 | Influenza virus B RNA [Presence] in Nasopharynx by NAA with probe detection | Influenza B |
| 80382-5 | Influenza virus A Ag [Presence] in Upper respiratory specimen by Rapid immunoassay | Influenza A |
| 80383-3 | Influenza virus B Ag [Presence] in Upper respiratory specimen by Rapid immunoassay | Influenza B |
| 80588-7 | Influenza virus A M gene [Presence] in Nasopharynx by NAA with probe detection | Influenza A |
| 80589-5 | Influenza virus A H1 HA gene [Presence] in Nasopharynx by NAA with probe detection | Influenza A/H1N1 |
| 80590-3 | Influenza virus A H3 HA gene [Presence] in Nasopharynx by NAA with probe detection | Influenza A/H3N2 |
| 82166-0 | Influenza virus A RNA [Presence] in Nasopharynx by NAA with non-probe detection | Influenza A |
| 82170-2 | Influenza virus B RNA [Presence] in Nasopharynx by NAA with non-probe detection | Influenza B |
| 85477-8 | Influenza virus A RNA [Presence] in Upper respiratory specimen by NAA with probe detection | Influenza A |
| 85478-6 | Influenza virus B RNA [Presence] in Upper respiratory specimen by NAA with probe detection | Influenza B |
| 92141-1 | Influenza virus B RNA [Presence] in Respiratory specimen by NAA with probe detection | Influenza B |
| 92142-9 | Influenza virus A RNA [Presence] in Respiratory specimen by NAA with probe detection | Influenza A |
| 92976-0 | Influenza virus B RNA [Presence] in Lower respiratory specimen by NAA with non-probe detection | Influenza B |
| 92977-8 | Influenza virus A RNA [Presence] in Lower respiratory specimen by NAA with non-probe detection | Influenza A |

**Table S2: ICD-10 codes associated with influenza.**

| **ICD-10 code** | **Description** |
| --- | --- |
| J09.x1 | Influenza due to identified novel influenza A virus with pneumonia |
| J09.x2 | Influenza due to identified novel influenza A virus with other respiratory manifestations |
| J09.x3 | Influenza due to identified novel influenza A virus with gastrointestinal manifestations |
| J09.x9 | Influenza due to identified novel influenza A virus with other manifestations |
| J10.00 | Influenza due to other identified influenza virus with unspecified type of pneumonia |
| J10.01 | Influenza due to other identified influenza virus with the same other identified influenza virus pneumonia |
| J10.08 | Influenza due to other identified influenza virus with other specified pneumonia |
| J10.1 | Influenza due to other identified influenza virus with other respiratory manifestations |
| J10.2 | Influenza due to other identified influenza virus with gastrointestinal manifestations |
| J10.83 | Influenza due to other identified influenza virus with otitis media |
| J10.89 | Influenza due to other identified influenza virus with other manifestations |
| J11.00 | Influenza due to unidentified influenza virus with unspecified type of pneumonia |
| J11.08 | Influenza due to unidentified influenza virus with specified pneumonia |
| J11.1 | Influenza due to unidentified influenza virus with other respiratory manifestations |
| J11.2 | Influenza due to unidentified influenza virus with gastrointestinal manifestations |
| J11.81 | Influenza due to unidentified influenza virus with encephalopathy |
| J11.89 | Influenza due to unidentified influenza virus with other manifestations |

**Table S3: Codes associated with influenza vaccines.**

| **Vaccine code** | **Description** |
| --- | --- |
| 146 | INFLUENZA VACCINE QUAD (FLUZONE/FLUARIX) (6 MONTHS AND OLDER)(PF) |
| 150 | IRB 17-005592 FLUZONE HIGH-DOSE OR STANDARD-DOSE INFLUENZA VACCINE |
| 155 | INFLUENZA VACCINE (FLUBLOK) (18 YEARS OR OLDER) (PF) |
| 161 | INFLUENZA VACCINE QUAD (FLUZONE) (6 MONTHS-35 MONTHS) (PF) |
| 185 | INFLUENZA VACCINE QV(FLUBLOK) (18 YEARS OR OLDER) (PF) |
| IIV | IRB- FLUARIX/FLUZONE INJ |
| IIV | flu vaccine qs 2015-16 (6mos+) Vial (PF) |
| IIV | flu vacc 2013-14 (36 mos+)(PF) Susp IM |
| IIV | flu vacc 2013-14 (6-35mos)(PF) Syringe I |
| IIV | flu vaccine 2012-13(3 yr+)(PF) Syringe I |
| IIV | flu vaccine qs 2014-15(6mos+) Susp IM |
| IIV | flu vacc 2013-14 (36 mos+)(PF) Syringe I |
| IIV | flu vacc ts 2013 (36 mos+)(PF) Susp IM |
| IIV | flu vac ts2013-14(36mo,up)(PF) Syringe I |
| IIV | flu vacc ts 2013 (6-35mos)(PF) Syringe I |
| IIV-HD | flu vacc ts 2014-15(65yr+)(PF) syringe I |
| IIV-HD | flu vacc ts 2013-14(65yr+)(PF) Syringe I |
| IIV-HD | flu vacc ts 2015-16(65yr+)(PF) syringe I |
| IIV3-HD | flu vacc ts2017-18 65yr up(PF) Syringe I |
| IIV3-HD | flu vacc ts2016-17 65yr up(PF) syringe I |
| IIV4 | flu vac qs 2015-2016 (PF) syringe IM |
| IIV4 | flu vaccine qs2016-17(6mos up) Susp IM |
| IIV4 | flu vaccine qs2017-18(6mos up) Susp IM |
| IIV4 | flu vac qs2016-17 36mos up(PF) Susp IM |
| IIV4 | flu vac qs2017-18 36mos up(PF) Syringe I |
| IIV4 | flu vac qs 2015-16(6-35mo)(PF) syringe I |
| IIV4 | flu vac qs2017-18 36mos up(PF) Susp IM |
| IIV4 | flu vac qs 2015-16(36mos+)(PF) Susp IM |
| IIV4 | flu vac qs 2016-17(6-35mo)(PF) syringe I |
| IIV4 | flu vac qs2016-17 36mos up(PF) syringe I |
| IIV4 | flu vac qs 2014-15(36mos+)(PF) syringe I |
| IIV4 | flu vac qs 2014-15(36mos+)(PF) Susp IM |
| IIV4 | flu vacc qs 2014 (6-35mos)(PF) syringe I |
| IIV4 | flu vac qs 2017-18(6-35mo)(PF) Syringe I |
| LAIV | flu vac qv live 2014 (2-49yrs) nasal spr |
| LAIV | flu vac 2013 (2-49yrs) Nasal Spray Syrin |
| LAIV | flu vac qv live 2015 (2-49yrs) nasal spr |
| null | Flublok (RIV3), 18-49yr |
| RIV3 | flu vac tv 2017(18yr up)rc(PF) Soln IM |
| RIV3 | flu vac tv 2014(18-49yr)rc(PF) Soln IM |
| RIV3 | flu vac tv 2015(18 yr+)rc(PF) Soln IM |
| RIV3 | flu vac tv 2013(18-49yr)rc(PF) Soln IM |
| RIV3 | flu vac tv 2016(18yr up)rc(PF) Soln IM |
| RIV4 | flu vac qv 2017(18yr up)rc(PF) Syringe I |

**Table S4: Viral symptoms and their synonyms used for augmented curation of clinical notes.**

| **Symptom** | **Synonyms/related entities identified in EHR** |
| --- | --- |
| Altered or diminished sense of taste or smell | anosmia,decrease in taste and smell,decrease in smell,change in smell,change in taste,bitter taste in her mouth,everything smells and tastes terrible,ageusia,lost her sense of smell,dysgeusia,change in smell and taste,loss of smell and taste,altered smell,change in his sense of taste and smell,altered sense of taste and smell,decreased taste,anosmia dysgeusia,decreased sense of taste,no sense of smell or taste |
| Chest pain/pressure | chest congestion,chest heaviness,chest pain,chest tightness,chest discomfort,pleuritic chest pain |
| Congestion | congestion,sinus congestion,congestion rhinorrhea,nasal congestion,head congestion,stuffy nose,sinus pressure |
| Conjunctivitis | watery eyes,watery eyes with redness,red eye,red eyes,pink eye |
| Cough | cough np p,non productive cough,cough,nonproductive cough,cough,dry cough |
| Dermatitis | rash |
| Diaphoresis | diaphoresis,night sweats,sweating,sweaty,sweats |
| Diarrhea | loose stools,diarrhea vomiting,loose stool,watery bm,watery diarrhea,diarrhea,soft stools |
| Dry mouth | dry mouth |
| Fatigue | fatigue,energy level is poor,fatigued,sleeping more than usual,lethargy |
| Fever / chills | subjective fever,fever,fevers,temp,felt warm,chills,tactile fever |
| Headache | ha,headache,has,sinus headache,headaches |
| Hemoptysis | blood tinged sputum,hemoptysis |
| Myalgia/Arthralgia | arthralgias,achy joints,back pain,ache,arthralgia,muscle aches,muscle discomfort,myalgia,sore neck,myalgias,body ache,body aches,aches and pains,joints became sore |
| Otitis | earache |
| Pharyngitis | tingly throat,throat discomfort,scratchy throat,sore throat |
| Productive cough | productive cough,cough productive,cough np p |
| Respiratory difficulty | dyspnea,tachypnea,tachypneic,lower respiratory symptoms,increased oxygen demands,labored breathing,dyspnea on exertion,sob,shortness of breath |
| Rhinitis | tickling in nose,sneezing,congestion rhinorrhea,runny nose,rhinorrhea,rhinitis,sniffles |
| Wheezing | wheezing |

**Table S5: Prevalences of COVID-19 + influenza, COVID-19, and influenza throughout the pandemic based on Mayo Clinic EHR data.** The “COVID-19 + Flu” cohort includes all individuals with a positive PCR test for SARS-CoV-2 and at least one of the following within 14 days: a positive PCR test for influenza, an ICD code for influenza, or a clinical note indicating a diagnosis of influenza. The “COVID-19” cohort includes all individuals with a positive PCR test for SARS-CoV-2. The “Flu” cohort includes all individuals with a positive PCR test for influenza.

| **Time period** | **COVID-19 + Flu cohort**  **(n = 120)**  Case count (%) | **COVID-19 cohort**  **(n = 197,364)**  Case count (%) | **Flu cohort**  **(n = 2,919)**  Case count (%) |
| --- | --- | --- | --- |
| March 12, 2020 - March 15, 2021 | 7 (5.8%) | 77,989 (39.5%) | 340 (11.6%) |
| March 16, 2021 - June 15, 2021 | 1 (0.8%) | 7,685 (3.9%) | 0 (0.0%) |
| June 16, 2021 - December 13, 2021 | 10 (8.3%) | 49,705 (25.2%) | 441 (15.1%) |
| December 14, 2021 - April 2, 2022 | 102 (85.0%) | 61,952 (31.4%) | 2138 (73.2%) |

**Table S6: Clinical characteristics of “COVID-19 + Flu” and “Overall COVID-19” cohorts at the Mayo Clinic during the Omicron era (December 14, 2021 - April 2, 2022).** The “COVID-19 + Flu” cohort includes all individuals with a positive PCR test for SARS-CoV-2 and at least one of the following within 14 days: a positive laboratory test for influenza, an ICD code for influenza, or a clinical note indicating a diagnosis of influenza. The “Overall COVID-19” cohort includes all individuals with a positive PCR test for SARS-CoV-2.

| **Clinical characteristic** | **COVID-19 + Flu cohort**  Case count (%) | **Overall COVID-19 cohort**  Case count (%) | **Relative risk**  **(95% CI)** |
| --- | --- | --- | --- |
| Total number of cases | 102 | 61,853 |  |
| Type of SARS-CoV-2 infection   - Primary infection - Re-infection | 94 (92.2%)  8 (7.8%) | 56,522 (91.4%)  5,331 (8.6%) | 1.01 [0.95, 1.06]  0.91 [0.50, 1.82] |
| COVID-19 vaccination status   - Unvaccinated - Partial - Full - Boosted | 67 (65.7%)  5 (4.9%)  30 (29.4%)  0 (0.0%) | 31,653 (51.2%)  2,729 (4.4%)  18,293 (29.6%)  9,178 (14.8%) | 1.28 [1.11, 1.47]***  1.11 [0.54, 2.73]  0.99 [0.74, 1.35]  0.00 [0.00, 0.52]*** |
| Initial COVID-19 vaccine type   - None - Janssen - Moderna - Pfizer/BioNTech | 67 (65.7%)  3 (2.9%)  16 (15.7%)  16 (15.7%) | 31,653 (51.2%)  1,718 (2.8%)  7,996 (12.9%)  20,486 (33.1%) | 1.28 [1.11, 1.47]***  1.06 [0.44, 3.43]  1.21 [0.80, 1.93]  0.47 [0.31, 0.75]*** |
| Flu vaccination status at time of SARS-CoV-2 infection   - Unvaccinated - Vaccinated | 90 (88.2%)  12 (11.8%) | 53,224 (86.0%)  8,629 (14.0%) | 1.03 [0.95, 1.10]  0.84 [0.52, 1.46] |
| Site   - Mayo Clinic - Arizona - Mayo Clinic - Florida - Mayo Clinic - Midwest | 0 (0.0%)  3 (2.9%)  99 (97.1%) | 7,205 (11.6%)  8,294 (13.4%)  46,354 (74.9%) | 0.00 [0.00, 0.66]***  0.22 [0.09, 0.71]***  1.29 [1.24, 1.34]*** |
| Sex   - Female - Male - Unknown / Non-binary | 45 (44.1%)  57 (55.9%)  0 (0.0%) | 32,697 (52.9%)  29,140 (47.1%)  16 (0.0%) | 0.83 [0.67, 1.04]  1.19 [1.00, 1.41]  0.00 [0.00, 301.81] |
| Race   - Asian - Black / African American - Native American - Native Hawaiian / Pacific Islander - White / Caucasian - Other - Unknown | 2 (2.0%)  8 (7.8%)  2 (2.0%)  0 (0.0%)  81 (79.4%)  7 (6.9%)  2 (2.0%) | 2,208 (3.6%)  3,052 (4.9%)  278 (0.4%)  115 (0.2%)  51,603 (83.4%)  1,950 (3.2%)  2,647 (4.3%) | 0.55 [0.20, 2.32]  1.59 [0.88, 3.18]  4.34 [1.57, 18.34]***  0.00 [0.00, 41.60]  0.95 [0.86, 1.05]  2.17 [1.16, 4.60]***  0.46 [0.17, 1.93] |
| Ethnicity   - Hispanic or Latino - Not Hispanic or Latino - Unknown | 10 (9.8%)  91 (89.2%)  1 (1.0%) | 4,516 (7.3%)  54,062 (87.4%)  3,275 (5.3%) | 1.34 [0.79, 2.48]  1.02 [0.95, 1.09]  0.19 [0.06, 1.35] |
| **Clinical characteristic** | **COVID-19 + Flu cohort**  Case count (%) | **Overall COVID-19 cohort**  Case count (%) | **Mann-Whitney U test p-value** |
| Age at time of positive PCR test for SARS-CoV-2 (in years)   - Mean: - Median: - Std dev: - IQR: | 24.9  18.3  19.1  (11.0, 38.4) | 39.9  38.5  21.3  (23.8, 56.3) | 8.9e-13*** |
| Elixhauser Comorbidity Index   - Mean: - Std dev: | 0.3  3.6 | 1.7  6.4 | 0.11 |

**Table S7: Clinical characteristics of matched “COVID-19 + Flu” and “COVID-19 mono-infection” cohorts at the Mayo Clinic.** The first cohort “Matched COVID-19 + Flu” includes all individuals with a confirmed co-infection of SARS-CoV-2 and influenza within 14 days along with at least 30 days of follow-up data. The second cohort “Matched COVID-19 mono-infection” includes all individuals who were selected as matched controls for the first cohort via propensity score matching with exact matching on the time period of infection (see Methods). Eligible matches for the second cohort include all individuals with a confirmed SARS-CoV-2 mono-infection (i.e. no co-infection with influenza) with at least 30 days of follow-up data.

| **Clinical characteristic** | **Matched**  **COVID-19 + Flu cohort**  Case count (%) | **Matched COVID-19 mono-infection cohort**  Case count (%) | **Relative risk**  **(95% CI)** |
| --- | --- | --- | --- |
| Total number of cases | 115 | 115 |  |
| Type of SARS-CoV-2 infection   - Primary infection - Re-infection | 108 (93.9%)  7 (6.1%) | 107 (93.0%)  8 (7.0%) | 1.01 [0.94, 1.08]  0.88 [0.34, 2.28] |
| Time of SARS-CoV-2 infection   - March 12, 2020 - March 15, 2021 - March 16, 2021 - June 15, 2021 - June 16, 2021 - December 13, 2021 - December 14, 2021 - March 3, 2022 | 7 (6.1%)  0 (0.0%)  10 (8.7%)  98 (85.2%) | 7 (6.1%)  0 (0.0%)  10 (8.7%)  98 (85.2%) | 1.00 [0.38, 2.66]  NA  1.00 [0.44, 2.26]  1.00 [0.90, 1.11] |
| COVID-19 vaccination status   - Unvaccinated - Partial - Full - Boosted | 80 (69.6%)  5 (4.3%)  30 (26.1%)  0 (0.0%) | 78 (67.8%)  6 (5.2%)  23 (20.0%)  8 (7.0%) | 1.03 [0.86, 1.22]  0.83 [0.28, 2.56]  1.30 [0.81, 2.08]  0.00 [0.00, 1.01] |
| Initial COVID-19 vaccine type   - None - Janssen - Moderna - Pfizer/BioNTech | 80 (69.6%)  3 (2.6%)  16 (13.9%)  16 (13.9%) | 78 (67.8%)  0 (0.0%)  5 (4.3%)  32 (27.8%) | 1.03 [0.86, 1.22]  NA  3.20 [1.18, 7.60]***  0.50 [0.30, 0.87]*** |
| Flu vaccination status at time of SARS-CoV-2 infection   - Unvaccinated - Vaccinated | 99 (86.1%)  16 (13.9%) | 101 (87.8%)  14 (12.2%) | 0.98 [0.89, 1.08]  1.14 [0.59, 2.19] |
| Site   - Mayo Clinic - Arizona - Mayo Clinic - Florida - Mayo Clinic - Midwest | 3 (2.6%)  5 (4.3%)  107 (93.0%) | 9 (7.8%)  15 (13.0%)  91 (79.1%) | 0.33 [0.11, 1.22]  0.33 [0.14, 0.91]***  1.18 [1.06, 1.31]*** |
| Sex   - Female - Male - Unknown / Non-binary | 51 (44.3%)  64 (55.7%)  0 (0.0%) | 50 (43.5%)  65 (56.5%)  0 (0.0%) | 1.02 [0.76, 1.36]  0.98 [0.78, 1.24]  NA |
| Race   - Asian - Black / African American - Native American - Native Hawaiian / Pacific Islander - White / Caucasian - Other - Unknown | 2 (1.7%)  8 (7.0%)  2 (1.7%)  0 (0.0%)  93 (80.9%)  8 (7.0%)  2 (1.7%) | 3 (2.6%)  5 (4.3%)  1 (0.9%)  0 (0.0%)  101 (87.8%)  1 (0.9%)  4 (3.5%) | 0.67 [0.14, 3.55]  1.60 [0.55, 4.38]  2.00 [0.22, 12.41]  NA  0.92 [0.82, 1.03]  8.00 [1.02, 31.54]***  0.50 [0.12, 2.55] |
| Ethnicity   - Hispanic or Latino - Not Hispanic or Latino - Unknown | 11 (9.6%)  103 (89.6%)  1 (0.9%) | 7 (6.1%)  102 (88.7%)  6 (5.2%) | 1.57 [0.63, 3.71]  1.01 [0.92, 1.11]  0.17 [0.04, 1.34] |
| **Clinical characteristic** | **Matched**  **COVID-19 + Flu cohort** | **Matched COVID-19 mono-infection cohort** | **Mann-Whitney U test p-value** |
| Age at time of positive PCR test for SARS-CoV-2 (in years)   - Mean: - Median: - Std dev: - IQR: | 26.6  20.0  20.3  (12.6, 40.7) | 26.5  21.7  20.1  (11.5, 35.1) | 0.95 |
| Elixhauser Comorbidity Index   - Mean: - Std dev: | 0.6  4.8 | 0.8  3.9 | 0.74 |
